# An initiator- and catalyst-free hydrogel coating process for 3D printed medical-grade poly(ε-caprolactone)

**DOI:** 10.3762/bjoc.17.136

**Published:** 2021-08-19

**Authors:** Jochen Löblein, Thomas Lorson, Miriam Komma, Tobias Kielholz, Maike Windbergs, Paul D Dalton, Robert Luxenhofer

**Affiliations:** 1 Polymer Functional Materials, Chair for Advanced Materials Synthesis, Institute for Functional Materials and Biofabrication, Department of Chemistry and Pharmacy, Julius-Maximilians-University Würzburg, Würzburg, Germany; 2 Institute of Pharmaceutical Technology and Buchmann Institute for Molecular Life Sciences, Goethe University, Frankfurt am Main, Germany; 3 Knight Campus for Accelerating Scientific Impact, University of Oregon, 1505 Franklin Blvd, Eugene, 97403 Oregon, USA; 4 Soft Matter Chemistry, Department of Chemistry and Helsinki Institute of Sustainability Science, Faculty of Science, University of Helsinki, 00014 Helsinki, Finland

**Keywords:** additive manufacturing, light-induced polymerization, self-initiated photografting and photopolymerization, surface-initiated polymerization, surface modification

## Abstract

Additive manufacturing or 3D printing as an umbrella term for various materials processing methods has distinct advantages over many other processing methods, including the ability to generate highly complex shapes and designs. However, the performance of any produced part not only depends on the material used and its shape, but is also critically dependent on its surface properties. Important features, such as wetting or fouling, critically depend mainly on the immediate surface energy. To gain control over the surface chemistry post-processing modifications are generally necessary, since it′s not a feature of additive manufacturing. Here, we report on the use of initiator and catalyst-free photografting and photopolymerization for the hydrophilic modification of microfiber scaffolds obtained from hydrophobic medical-grade poly(ε-caprolactone) via melt-electrowriting. Contact angle measurements and Raman spectroscopy confirms the formation of a more hydrophilic coating of poly(2-hydroxyethyl methacrylate). Apart from surface modification, we also observe bulk polymerization, which is expected for this method, and currently limits the controllability of this procedure.

## Introduction

Additive manufacturing, commonly referred to as three-dimensional (3D) printing, is an approach to create physical objects using layer-by-layer [1] or voxel-by-voxel fabrication [2]. 3D printed materials can be used for a broad spectrum of applications, including medical devices where implants can be personalized to improve outcomes in patients [3]. Here, compliance to the regulatory pathway [4–5] is important, which favors solvent-free processing technologies of medical-grade raw materials. Solvent-free 3D printing approaches such as electron beam melting [6], micro-extrusion [7] and selective laser sintering have all been used to fabricate clinical implants [6–7].

Other emerging 3D printing technologies can also fit the prerequisites described above, including melt electrowriting (MEW) [8]. This technology is based on the direct-writing of ultra-fine (800 nm to 10 µm) and micro-scale filaments onto a collector with repeat fiber deposition to build up a micro-scale 3D object, with build heights in the range of several millimeters [9–11]. In MEW, the polymer jet is electrostatically drawn from a nozzle, resulting in a considerable reduction of the final fiber diameter. The direct-writing onto a flat collector or on a rotating/translating mandrel enables the printing of flat [11–12] or tubular [13] scaffolds, respectively.

**Scheme 1:**
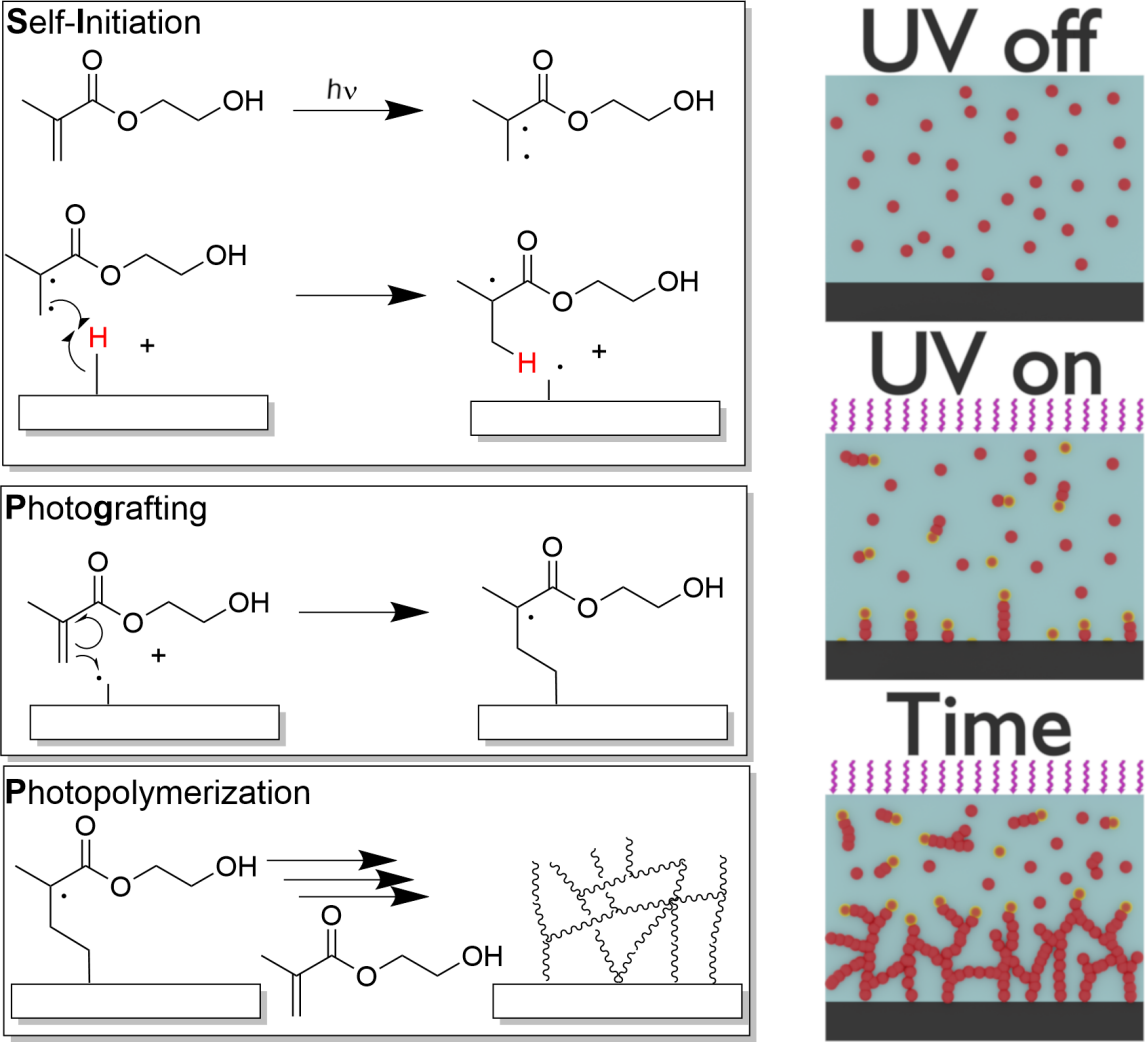
Schematic representation of the self-initiated photografting and photopolymerization (SIPGP) of 2-hydroxyethyl methacrylate (HEMA) including intersystem crossing from singlet to triplet state which is mandatory for the formation of a biradical (left). Cartoon how SIPGP leads to surface-initiated polymerization and bulk polymerization (monomer red, active species red/yellow halo) (right).

While medical-grade poly(ε-caprolactone) (PCL) is the most commonly used material for MEW, due to its favorable thermal and mechanical properties, cytocompatibility, biodegradability, and good printing properties [8,14–15], it is a hydrophobic polymer and immersion into fluids can result in air bubble capture within the scaffold structure, biofouling and non-specific cell interactions [16]. Protein adsorption and denaturation on the hydrophobic surface can lead to fibrous encapsulation of medical implants [17–18]. Accordingly, it would be beneficial for PCL implants to have a hydrophilic, non-fouling surface while avoiding factors that complicate the regulatory path to the clinic. So far, the wettability could be improved by a variety of methods like plasma treatment, NaOH hydrolysis treatment or a micro deposition system. This study outlines a potential approach to coat medical-grade PCL with a thin hydrogel that requires no initiator or catalyst – just a deoxygenated aqueous monomer solution and UV light. We demonstrate this surface modification using a hydrogel, poly(2-hydroxyethyl methacrylate) (PHEMA) [19]. However, this approach should have broad utility for a spectrum of monomers and macromonomers susceptible to radical polymerization onto (almost) any surface featuring C–H bonds. This hydrogel coating is achieved with self-initiated photografting and photopolymerization (SIPGP, Scheme 1).

SIPGP is a simple, solvent-free bulk/surface photografting first introduced by Deng et al. grafting maleic anhydride [20] and styrene [21] onto low-density poly(ethylene) films. Later, this was extended for other monomers and many other substrates including ultra-nanocrystalline diamond, graphene and silicon carbide [22–30]. Even bottle-brush brushes could be prepared via SIPGP on various substrates for different applications [31–33].

## Results and Discussion

**Figure 1:**
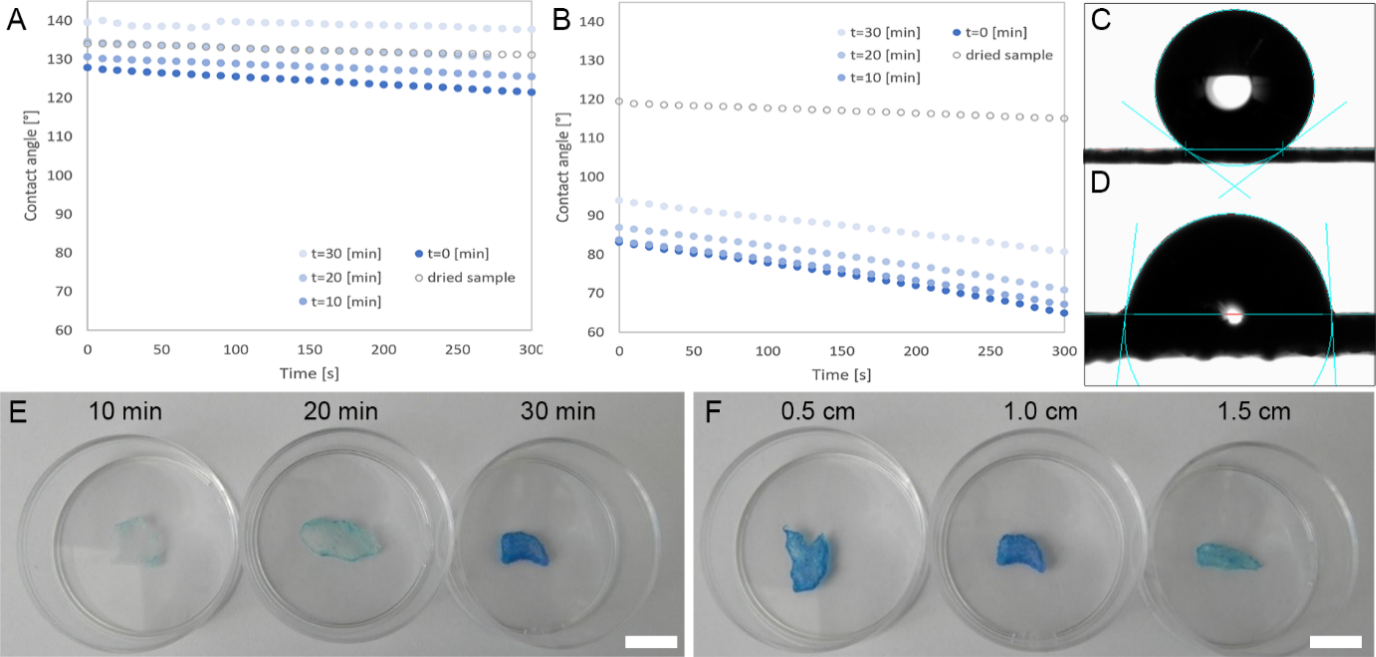
A) Graph showing change in the static contact angle with time on a pristine PCL scaffold with a 500 µm hatch spacing and B) on a SIPGP-coated scaffold with a 500 µm hatch spacing. The measured samples were immersed in water for different periods of time *t* = 0, 10, 20, 30 [min]. C) Photograph of a 2 µL water drop on a pristine PCL scaffold immediately after droplet deposition and D) on a SIPGP-modified scaffold immediately after droplet deposition. E and F) toluidine blue staining of PCL scaffolds coated with PHEMA via SIPGP showing the variation with E) the UV-reaction time and F) the distance between light source and substrate. Scale bar for E and F is 1 cm.

Here, we introduce SIPGP for the coating of 3D printed PCL microfiber scaffolds, investigate the range of suitable parameters and characterize the change in the properties of the coated scaffolds. PCL scaffolds with a hatch spacing of either 150 µm or 500 µm were fabricated, with the fiber diameter being 12.1 ± 3.3 µm. The experimental setup consisted of a closed chamber, which was equipped with a valve for argon supply and a slot for the UV lamp (Supporting Information File 1, Figure S1). Initially, static conditions were used to determine the influence of exposure time, distance between light source and substrate on the PHEMA coating and monomer concentration (Supporting Information File 1, Figure S2). As the next step, scaffolds were investigated with respect to their wettability. In order to simulate aqueous conditions in cell culture medium or after implantation, samples were immersed in water for 0, 10, 20 and 30 min (Figure 1A and Figure 1B; *t* = 0, 10, 20, 30 [min]) before analysis. Based on contact angle measurements, the wettability of PCL scaffolds notably changed after SIPGP modification (Figure 1). The hydrophobicity of untreated PCL MEW scaffolds (500 µm hatch spacing) is also affected by the macrostructure of the hatches, with a high contact angle of at least 127.9° (Figure 1A, *t* = 0 [min]) (compared to 66° reported for a smooth PCL film [34]) that remains almost unchanged over time. SIPGP-modified PCL scaffolds, however, revealed a significantly lower initial contact angle. For 500 µm hatch spacing, an initial contact angle of 83.2° (*t* = 0 [min]) was measured and decreased until a final value of 64.9° after 300 s (Figure 1B). The decrease in contact angle is more rapid compared to the uncoated PCL scaffold, because further water is adsorbed by the hydrophilic PHEMA coating, resulting in enhanced wettability. However, both graphs also show a time-dependent wettability decrease in the first 30 min after the withdrawal from immersion which might be in consequence of a hydration loss through water evaporation. Interestingly, this tendency is only followed by the dried PHEMA-coated sample and not by the dried pristine PCL scaffold. An explanation for this observation might be PHEMA´s ability to absorb water as well as the relative indistinct hydration state of PCL compared to PHEMA when fully hydrated.

Toluidine blue staining was used as a rapid, colorimetric readout as it selectively binds to PHEMA. Results for three negative controls (cleaned scaffold, scaffold irradiated without monomer solution, and scaffold immersed in monomer solution without irradiation) can be found in Supporting Information File 1, Figure S3, and have no staining. The irradiation time was varied between 10 min, 20 min, and 30 min (Figure 1E) and the intensity of the blue coloration is proportional to reaction time resulting in a complete coating of the PCL fibers after 30 min. The distance between the UV source and the scaffold was varied from 0.5 cm, 1.0 cm, and 1.5 cm (Figure 1F) and, as the intensity decreases inversely in proportion to the square of the distance, a larger working distance should result in less pronounced coating.

**Figure 2:**
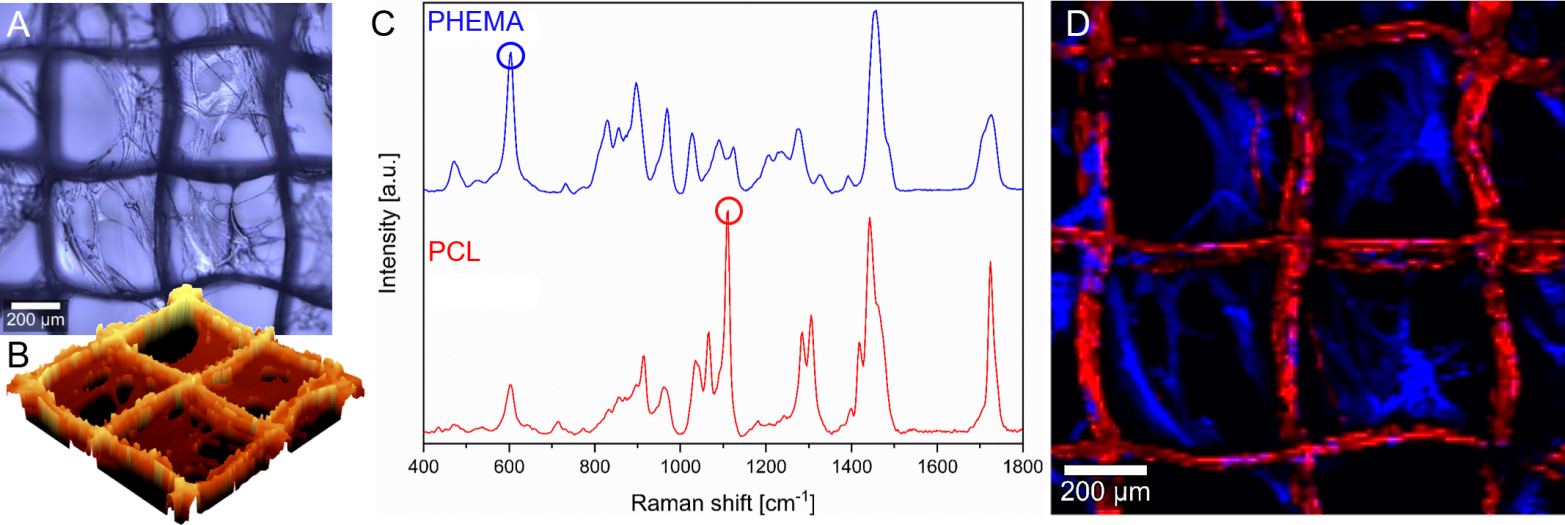
A) Optical photograph of an SIPGP-coated sample. B) 3D topography reconstruction of the SIPGP-coated scaffold. C) Raman spectra depicting the fingerprint region of PHEMA and PCL with the two significant peaks of PHEMA (829 cm^−1^) and PCL (1111 cm^−1^) marked with circles. (D) Shows these peaks as False-color Raman image of PCL fibers (red) and PHEMA coating (blue).

Wang and Brown reported a polymerization conversion of 90% after 50 s for HEMA (0.30 mm ± 0.04 mm) [22] which is significantly faster than the values investigated in the present study. However, the authors used UV-C light with an intensity of 0.024 W/cm^2^ compared to UV-A light with a calculated intensity of 0.079 W/cm^2^ at a distance of 1.0 cm used in the present study. After SIPGP, a thin, heterogeneous material can be seen spanning large proportion of the pores (Figure 2A and Figure 2B), however, this morphology is highly irregular. A change in wetting behavior could be demonstrated, and toluidine blue staining indicates successful SIPGP of HEMA on PCL scaffolds. However, to prove that this material is indeed PHEMA, we performed confocal Raman microscopy on the samples, which allows a spatially resolved polymer identification based on chemical functionalities. A unique peak at 1111 cm^−1^ was identified and is attributed to backbone stretching of aliphatic chains [ν(C–C)] in PCL. Also, a characteristic band at 829 cm^−1^ was detected which can be assigned to PHEMA and refers to the symmetrical stretching vibration [ν(C–O–C)] of ester groups (Figure 2C, blue and red circles). With Raman imaging and the corresponding spectra, the presence of PHEMA on the PCL MEW scaffolds was clearly verified (Figure 2C and Figure 2D), especially regarding the material spanning the boxes.

**Figure 3:**
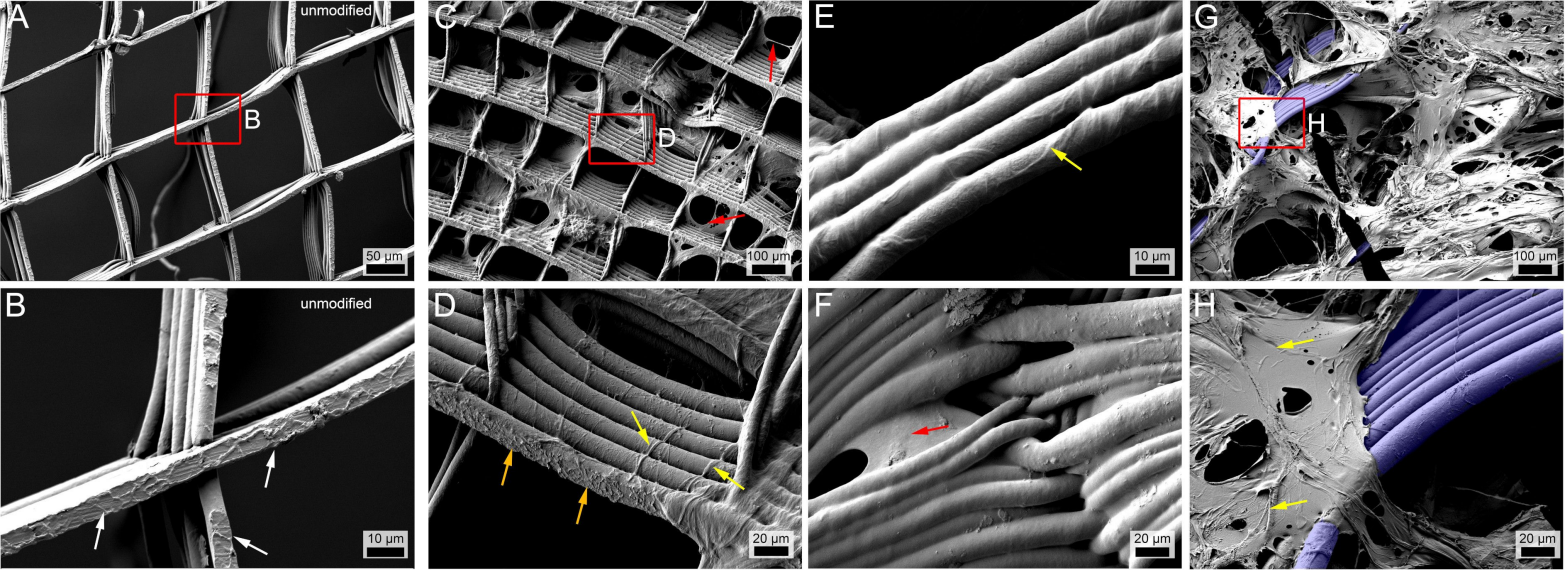
A) SEM image of pristine, uncoated PCL MEW scaffolds with a hatch spacing of 150 µm × 200 µm and inset (B), where the white arrows show embossing of the first printed layer on the bottom of the scaffold. C) PHEMA coated scaffolds (prominent spots of PHEMA coating marked with red arrows) of the same hatch spacing, irradiated from a distance of 1 cm for 30 min with a wavelength of 365 nm with inset shown in (D), where the yellow arrows point at PHEMA strands, in contrast to the irregularities caused by the printing (orange arrows). E and F) SEM images of scaffolds with a hatch spacing of 500 µm under the same conditions as C). The yellow arrow in (E) points at a border line of PHEMA and the red arrow in (F) at pure PHEMA in between fibers. G) SEM image of a scaffold with a hatch spacing of 500 µm under the same conditions as C), but freeze dried, with inset shown in (H), where the yellow arrows point at irregularities within the PHEMA coating. False colored blue for G and H indicates the exposed PCL fibers in the PHEMA-filled MEW scaffolds.

Unfortunately, the high PCL signal intensity covers most of the signals originating from the thin PHEMA coating directly deposited on fibers (Supporting Information File 1, Figure S4; a 3D Raman reconstruction is provided in Supporting Information File 2). Scanning electron microscopy (SEM) corroborates this fiber coating and corroborates the inhomogeneous distribution of PHEMA inside the boxes (Figure 3). Compared to the pristine scaffold (Figure 3A and Figure 3B), the PHEMA coat can be clearly distinguished both as a thin coat on the fibers as well as in form of films between fibers and spanning boxes (Figure 3C). On unmodified scaffolds, rough patches on the bottom-most fibers are discernible (Figure 3B, white arrows), which originate from the contacting of the first fibers with the collector. After coating, these rough patches are covered by more smooth material, but only partially (Figure 3D, orange arrows). The coating is clearly irregular, even where fibers are fully coated, with seemingly thicker strands of PHEMA covering the PCL scaffold (Figure 3C and Figure 3E, yellow arrow). We assume that these thicker strands may originate from UV-induced HEMA polymerization in solution which are then deposited on the scaffold. Interestingly, profound webbing of PHEMA between the fibers can be observed (Figure 3F, red arrow), but also rather inhomogeneous membranous depositions on larger sections of the scaffolds (Figure 3G and Figure 3H).

Even though MEW has been carried out predominantly using PCL, other non-conductive polymers like water-soluble poly(2-ethyl-2-oxazoline) (PEtOx) [35], polypropylene (PP) [36–37] photo-cross-linkable and biodegradable poly(ʟ-lactide-*co*-ε-caprolactone-*co-*acryloyl carbonate) [38], or thermoplastic elastomers [39] have successfully been processed via MEW [40]. Interestingly, PCL frames made with MEW were used to build up soft network composites [41–43] with outstanding mechanical properties, also with weak matrices of interest for tissue engineering applications [44].

## Conclusion

SIPGP is an interesting complementary technique to modify the surface of MEW printed scaffolds, which may be required for suitable interaction with cells or other materials. SIPGP does not require special equipment other than a UV lamp or special reagents. As a proof-of-concept, we demonstrated the feasibility using HEMA as monomer, but essentially any monomer polymerizable by free radical polymerization should be feasible. At this point, the coating of the fibrous scaffolds is rather inhomogeneous, as the surface-initiated polymerization is confounded by polymerization in bulk. Therefore, we are currently working on a slightly modified experimental setup, which utilizes a flow setup, constantly removing the bulk materials and facilitating a more homogeneous surface coating.

## Supporting Information

File 1 Experimental section and additional images.

File 2 3D Raman reconstruction.
