# Supplementary material for: An initiator- and catalyst-free hydrogel coating process for 3D printed medical-grade poly(ε-caprolactone)
Source: Beilstein J Org Chem. 2021 Aug 19;17:2095–101. doi: 10.3762/bjoc.17.136 (PMC8381808; doi:10.3762/bjoc.17.136)
Supplement: File 1 — Experimental section and additional images. [file Beilstein_J_Org_Chem-17-2095-s001.pdf]

BEILSTEIN JOURNAL OF ORGANIC CHEMISTRY

Supporting Information

for

An initiator- and catalyst-free hydrogel coating process for 3D printed medical-grade poly(ε-caprolactone)

Jochen Löblein, Thomas Lorson, Miriam Komma, Tobias Kielholz, Maike Windbergs, Paul D. Dalton and Robert Luxenhofer

Beilstein J. Org. Chem. **2021,** *17,* 2095–2101. doi:10.3762/bjoc.17.136

Experimental section and additional images

License and Terms: This is a supporting information file under the terms of the Creative Commons Attribution License (https://creativecommons.org/licenses/by/4.0). Please note that the reuse, redistribution and reproduction in particular requires that the author(s) and source are credited and that individual graphics may be subject to special legal provisions.
The license is subject to the *Beilstein Journal of Organic Chemistry* terms and conditions: (https://www.beilstein-journals.org/bjoc/terms)

Experimental

Materials

Poly(ε-caprolactone) PCL was received in medical grade from *Purac* and was stored at 8 °C under inert atmosphere. Using MEW scaffolds in the size of 5 cm × 5 cm with a hatch spacing of either 150 µm or 500 µm and 10 stacked up layers were printed. 2-Hydroxyethyl methacrylate (HEMA) was purchased from Sigma-Aldrich GmbH with a purity of >99% with 50 ppm monomethyl ether hydroquinone as inhibitor and was stored at 8 °C under the exclusion of light. Before usage HEMA was distilled. Toluidine blue was purchased from Sigma-Aldrich GmbH and was stored at room temperature. For usage a 0.05% toluidine blue solution was produced from 50.5 mg toluidine blue and 100 mL of distilled water.

Preparation of the samples

The HEMA solutions were prepared in the volume ratio HEMA 9:11 Millipore water. The PCL samples used were cut into small pieces and washed in Millipore water for 30 min before usage. Subsequently, they were put into a reaction chamber consisting of a box with an argon inlet and the UV-lamp VL-4LC by Vilber Lourmat. A stack of glass slides was used to alter the distance between the sample and the light source. The light source was a UV-light tube with 4 W and 365 nm wavelength. On top of the samples 1–2 mL of monomer solution gets added. The reaction then is performed under argon atmosphere. After completion the samples were rinsed 5× with Millipore water for 1 min. For visualization of the formed hydrogel the samples were put into the toluidine blue solution.

Contact Angle

Contact angle measurements were performed with an OCA 15LJ (DataPhysics Instruments GmbH). Scaffolds were immersed in distilled water for 7 days and withdrawn prior to measurement. Measurement was conducted directly after withdrawal from water as well as 10, 20, and 30 min later. Remaining adhesive water was gently swabbed away and specimens were mounted in a holder. The applied drop volume was 2 µL of distilled water. Subsequently, specimens were dried in a desiccator at ambient temperature and served as a reference.

Confocal Raman microscopy

Untreated scaffolds were fixed on a calcium fluoride slide with metal clamps. Raman spectra were acquired using a confocal Raman microscope (alpha 300R+, WITec GmbH) with a 50× objective (N.A. 0.8, Epiplan Neofluar, Zeiss, Germany) and a 532 nm diode laser (38.6 mW power before the objective). As the surface of the scaffolds was structured, topography maps of the sample surface were assessed prior to acquisition of Raman spectra enabling the visualization of the entire sample surface. Raman spectra were recorded every 10 µm in x and y direction with an integration time of 1 s. After removal of cosmic ray-based signals and background subtraction, spectra were post-processed by cluster analysis.

SEM imaging

The SEM images were taken by the GEMINI Crossbeam 340 by Zeiss. The samples were sputtered with 4 nm of platinum as preparation and 100 separate fibers were used for diameter measurements.

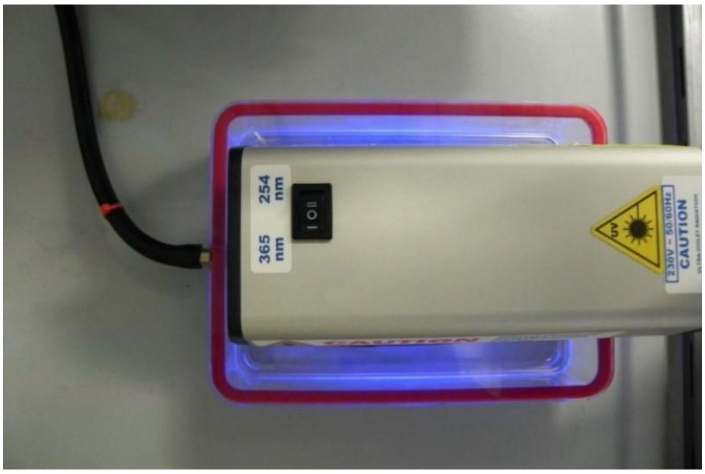

Figure S1: Experimental setup including valve for argon, reaction chamber and UV-lamp.

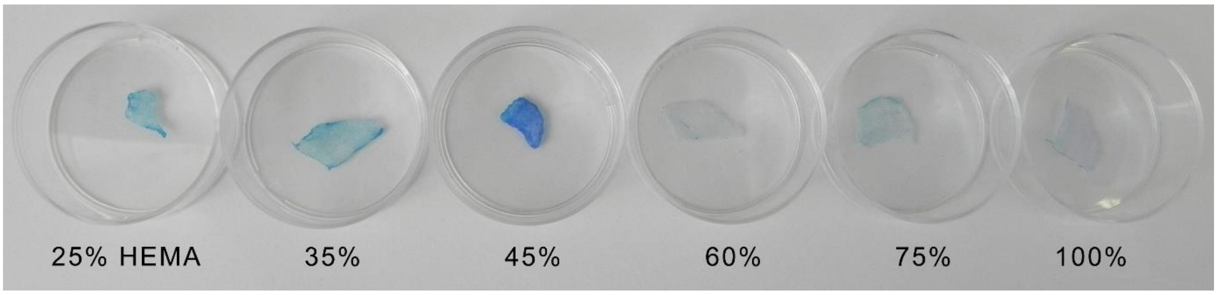

Figure S2; Staining of scaffolds after SIPGP using different HEMA/water ratios.

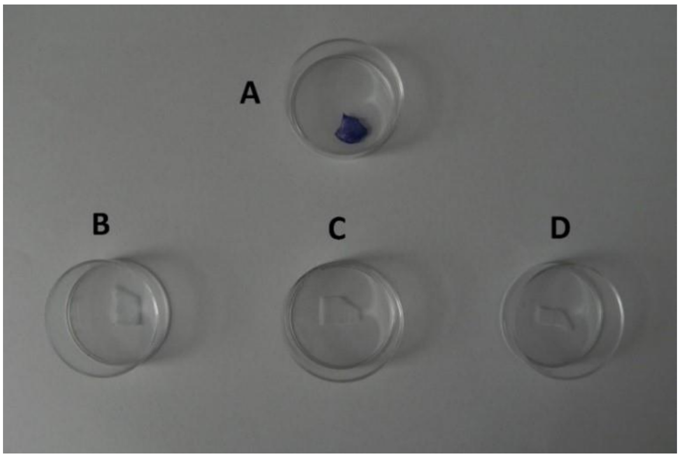

Figure S3: Staining of scaffolds with B, C and D being the negative controls.

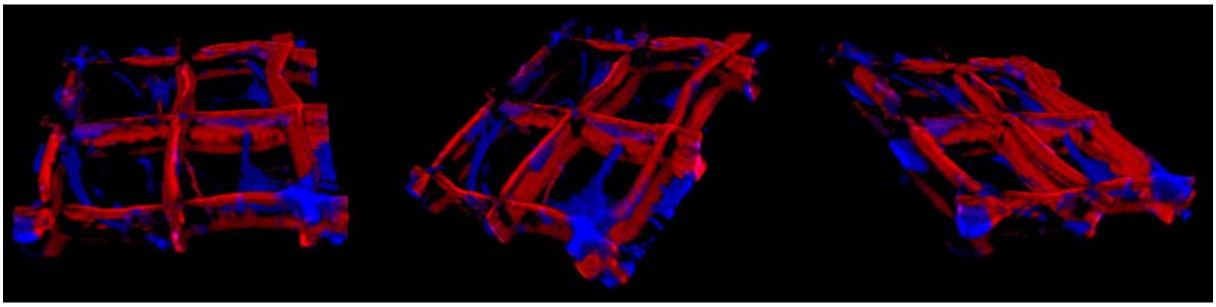

Figure S4: Images with different angles of a 3D Raman image reconstruction taken from Supporting Information File 2 showing PCL fibers (red) and PHEMA (blue).
